# Gut-microbiome-based predictive model for ST-elevation myocardial infarction in young male patients

**DOI:** 10.3389/fmicb.2022.1031878

**Published:** 2022-12-01

**Authors:** Mingchuan Liu, Min Wang, Tingwei Peng, Wenshuai Ma, Qiuhe Wang, Xiaona Niu, Lang Hu, Bingchao Qi, Dong Guo, Gaotong Ren, Jing Geng, Di Wang, Liqiang Song, Jianqiang Hu, Yan Li

**Affiliations:** ^1^Department of Cardiology, Tangdu Hospital, The Fourth Military Medical University, Xi’an, China; ^2^Department of Pulmonary and Critical Care Medicine, Xijing Hospital, The Fourth Military Medical University, Xi'an, China

**Keywords:** STEMI, gut microbiome, young male patients, prediction model, 16S rRNA

## Abstract

**Background:**

ST-segment elevation myocardial infarction (STEMI) in young male patients accounts for a significant proportion of total heart attack events. Therefore, clinical awareness and screening for acute myocardial infarction (AMI) in asymptomatic patients at a young age is required. The gut microbiome is potentially involved in the pathogenesis of STEMI. The aim of the current study is to develop an early risk prediction model based on the gut microbiome and clinical parameters for this population.

**Methods:**

A total of 81 young males (age < 44 years) were enrolled in this study. Forty-one young males with STEMI were included in the case group, and the control group included 40 young non-coronary artery disease (CAD) males. To identify the differences in gut microbiome markers between these two groups, 16S rRNA-based gut microbiome sequencing was performed using the Illumina MiSeq platform. Further, a nomogram and corresponding web page were constructed. The diagnostic efficacy and practicability of the model were analyzed using K-fold cross-validation, calibration curves, and decision curve analysis (DCA).

**Results:**

Compared to the control group, a significant decrease in tendency regarding α and β diversity was observed in patients in the case group and identified as a significantly altered gut microbiome represented by *Streptococcus* and *Prevotella*. Regarding clinical parameters, compared to the control group, the patients in the case group had a higher body mass index (BMI), systolic blood pressure (SBP), triglyceride (TG), alanine aminotransferase (ALT), and aspartate aminotransferase (AST) and low blood urea nitrogen (BUN). Additionally, BMI and SBP were significantly (p<0.05) positively correlated with *Streptococcus* and *[Ruminococcus]*. Further, BMI and SBP were significantly (p<0.05) negatively correlated with *Prevotella* and *Megasphaera*. A significant negative correlation was only observed between *Prevotella* and AST (*p* < 0.05). Finally, an early predictive nomogram and corresponding web page were constructed based on the gut microbiome and clinical parameters with an area under the receiver-operating characteristic (ROC) curve (AUC) of 0.877 and a C-index of 0.911. For the internal validation, the stratified *K*-fold cross-validation (*K* = 3) was as follows: AUC value of 0.934. The calibration curves of the model showed good consistency between the actual and predicted probabilities. The DCA results showed that the model had a high net clinical benefit for use in the clinical setting.

**Conclusion:**

In this study, we combined the gut microbiome and common clinical parameters to construct a prediction model. Our analysis shows that the constructed model is a non-invasive tool with potential clinical application in predicting STEMI in the young males.

## Introduction

ST-segment elevation myocardial infarction (STEMI) is the most severe coronary artery disease (CAD), with a high risk of complications and even death. Recent studies have shown a significant increase in the incidences of STEMI and the rate of hospitalization in young males <55 years of age, which indicated the necessity to investigate this population (Zhang et al., 2016). Compared to elderly patients, young male patients with ischemic heart disease present different clinical characteristics, such as a higher proportion of single-vessel lesions, more percutaneous interventions (PCI), and an increased risk of adverse outcomes post-hospitalization (Bangalore et al., 2012; Gupta et al., 2014). Moreover, the absence of traditional risk factors such as diabetes, hypertension, and hyperlipidemia in these patients indicates a distinct disease pattern (Wittlinger et al., 2020) and the obscurity of the underlying pathophysiology. Therefore, early diagnosis of these patients is crucial for better outcomes. However, the lack of characteristic risk factors for screening STEMI in the young population is a major challenge in timely diagnosis and intervention.

Studies have shown that the gut microbiome plays an important role in maintaining normal physiological functions in the human body (Lozupone et al., 2012; Pietroiusti et al., 2016). Disturbances in the gut microbiota have been associated with multiple human diseases, including CAD (Liu et al., 2019). Previous studies have reported that *Bacteroides* and gut microbiome-derived metabolites such as trimethylamine N-oxide (TMAO) mediated atherosclerosis formation and are associated with the incidence of CAD (Koeth et al., 2013; Yoshida et al., 2018). The combination of clinical characteristics and gut microbiome could significantly enhance the diagnostic efficacy of CAD (Zheng et al., 2020). These findings are indicative of the potential role of the microbiome in STEMI in young males. However, studies on this issue are rare, and details regarding the relationship between the gut microbiome and STEMI in young men remain elusive.

The aim of the current study is to investigate the alteration in the gut microbiome in young male patients with STEMI. We investigated the correlation between the gut microbiome and clinical parameters. Further, we developed an early risk prediction model based on the gut microbiome and the clinical parameters and performed internal validation. Our results may provide novel insights into the pathogenesis and prevention of STEMI in young males.

## Materials and methods

### Study design and population

A total of 81 young males were enrolled in this case–control study. The case group comprised 41 young males diagnosed with STEMI at the department of cardiology of the Tangdu Hospital of Air Force Medical University. The inclusion criteria were as follows: (1) markedly acute chest pain in the last 24 h. (2) ST-segment elevation or depression or pathological Q wave detected by electrocardiogram. (3) elevation in serum cardiac troponin (cTnI) with values above the 99^th^ percentile upper reference limit. (4) intraluminal obstruction revealed by coronary angiography. (5) age between 18 and 44 years old. The control group comprised 40 age-matched males with negative results per coronary angiography. In both groups, individuals with prior medical conditions like malignancy, severe liver or kidney disease, autoimmune disease, gastrointestinal disease, >3 days of antibiotic use in the past 3 months or an abnormal stool morphology, such as diarrhea and dry stools were excluded from the study. The study was approved by the ethics committee of Tangdu Hospital of Air Force Medical University. All patients provided written informed consent to participate in the study.

### Baseline characteristics and biochemical tests

The demographic characteristics like age, height, weight, smoking, and alcohol habits, as well as the past medical history of the patients retrieved from the medical record system of the Tangdu Hospital of Air Force Medical University. Five milliliter of venous blood was drawn from all patients in the morning on the second-day post-hospitalization after overnight fasting to test the liver and kidney function indices, lipid and blood glucose analysis at the Center Laboratory Medicine of Tangdu Hospital.

### Collection and storage of stool samples

All subjects were asked to retain their stool sample in the morning post-hospitalization. The samples were collected with sterile stool collectors by professionally trained personnel. The samples were transported to the laboratory with 2 h of sample collection and stored at −80°C.

### DNA extraction and 16S rRNA sequencing

The bacterial DNA was isolated from the patients’ fecal samples using the bead-beating method described previously (Yang et al., 2021). The extracted DNA was used as a template to amplify the 16S rRNA gene V3-V4 region by polymerase chain reaction (PCR). The sequencing libraries were generated using a TruSeq^®^ DNA PCR-Free sample preparation kit (Illumina, CA, United States), and indexing codes were added per the manufacturer’s instructions. The quality of the library was assessed using the Qubit @2.0 fluorometer (Thermo Fisher Scientific, MA, United States) and the Bioanalyzer 2,100 system (Agilent, CA, United States). The validated libraries were sequenced using the Illumina MiSeq platform (Personalbio, Shanghai, China) to generate 2 × 300 bp side reads per the manufacturer’s instructions.

### Sequencing analysis

Microbiome bioinformatics were performed with QIIME2 2019.4 with slight modification according to the official tutorials (https://docs.qiime2.org/2019.4/tutorials/). Briefly, raw sequence data were demultiplexed using the demux plugin following by primers cutting with cutadapt plugin. Sequences were then quality filtered, denoised, merged and chimera removed using the DADA2 plugin. Non-singleton amplicon sequence variants (ASVs) were aligned with mafft and used to construct a phylogeny with fasttree2. All samples included in these analyses included at least 2,800 reads. Exact read numbers per sample are included in Supplementary Table S2. ASV-level alpha diversity indices, such as Chao1 richness estimator, Observed species, Shannon diversity index, Simpson index, were calculated using the ASV table in QIIME2, and visualized as box plots. ASV-level ranked abundance curves were generated to compare the richness and evenness of ASVs among samples. Beta diversity analysis was performed to investigate the structural variation of microbial communities across samples using Jaccard metrics, Bray-Curtis metrics and UniFrac distance metrics and visualized *via* principal coordinate analysis (PCoA), nonmetric multidimensional scaling (NMDS) and unweighted pair-group method with arithmetic means (UPGMA) hierarchical clustering. Principal component analysis (PCA) was also conducted based on the genus-level compositional profiles. The significance of differentiation of microbiota structure among groups was assessed by PERMANOVA (Permutational multivariate analysis of variance), ANOSIM (Analysis of similarities), Permdisp using QIIME2. Taxonomy was assigned to ASVs using the classify-sklearn naïve Bayes taxonomy classifier in feature-classifier plugin against the Greengenes (Release 13.8, http://greengenes.secondgenome.com/). LEfSe (Linear discriminant analysis effect size) was performed to detect differentially abundant taxa across groups using the default parameters. OPLS-DA (Orthogonal Partial Least Squares Discriminant Analysis) was also introduced as a supervised model to reveal the microbiota variation among groups, using the R package “muma.” Random forest analysis was applied to discriminating the samples from different groups using QIIME2 with default settings.

### Construction and validation of diagnostic models

A univariate and multivariate logistic regression analysis with forward stepwise selection was performed to identify independent variables with a *p* < 0.05 and were entered into the final model. Next, the three diagnostic models, i.e., clinical, microbiome, and combined models, were established. The accuracy of each variable for predicting STEMI was confirmed using receiver-operating characteristic (ROC) curve analysis. The nomogram was used to visualize the risk of STEMI based on the selected clinical parameters and gut microbiota. The internal model validation was performed by the bootstrap resampling method with 500 resamples. 70% of the population was used as the training set, and the remaining 30% of the population was used for K-fold cross-validation (*K* = 3). The area under the curve (AUC) was used to evaluate the accuracy and generalizability of the model. The consistency between the predicted and the observed results were evaluated by plotting the calibration curve. The decision-making curve analysis (DCA) was used to evaluate clinical practicability. Finally, a dynamic nomogram web page with an interactive interface was constructed for clinical use.

### Statistical analysis

The student’s *t-*test or Wilcoxon rank-sum test was used for continuous variables, and the χ^2^ test was used for categorical variables to compare the distribution of baseline characteristics of the study population. LDA effect size (LEfSe) statistical analysis and random forest analysis were performed to screen for the differential gut microbiome between the two groups. The correlation heatmap between the microbiome and clinical variables was constructed based on Spearman’s rank correlation analysis. The association between STEMI and clinical parameters as well as microbial features was determined using logistics regression analysis. *p* < 0.05 was considered statistically significant. All statistical analyses were performed using R studio software (version 4.2.0).

## Results

### Baseline characteristics of the patients in the two groups

A total of 81 age and gender-matched patients were included in the study, of which 41 young male patients with STEMI were included in the case group, and 40 non-CHD males were included in the control group. As is shown in Table 1, compared to the control group, the patients in the case group had a significantly (*p* < 0.05) high body mass index (BMI), systolic blood pressure (SBP), triglyceride (TG), alanine aminotransferase (ALT), and aspartate aminotransferase (AST). However, a significantly low blood urea nitrogen (BUN) (*p* < 0.05) was observed in the patients in the case group compared to the control group. No statistically significant differences were observed between the two groups in the following parameters: age, beat per minute (BPM), hypertension (HTN), comorbidity, ejection fraction (EF), fasting blood glucose (FBG), total cholesterol (TC), high-density lipoprotein cholesterol (HDL-C), low-density lipoprotein cholesterol (LDL-C), creatinine (Cr), uric acid (UA), and habits like drugs, smoking, and alcohol.

**Table 1:** Demographic and clinical characteristics of the control and STEMI groups.

Variables	Control(*n* = 40)	STEMI(*n* = 41)	*p-*values
Age(years)	35.65 ± 6.52	38.27 ± 5.43	0.053
BMI(kg/m^2^)	24.47 ± 2.08	26.47 ± 2.57	<0.001
SBP (mmHg)	120.73 ± 15.33	135.90 ± 26.59	0.002
DBP (mmHg)	84.98 ± 16.01	80.63 ± 12.23	0.176
BPM	78.80 ± 12.00	78.54 ± 12.02	0.502
HTN (%)	8/40	16/41	0.061
HTN Drugs (%)	7/40	11/41	0.313
DM (%)	1/40	4/41	0.175
DM Drugs(%)	1/40	4/41	0.175
Smoking (%)	19/40	25/41	0.224
Alcohol (%)	9/40	12/41	0.487
Comorbidity (%)	5/40	7/40	0.562
EF (%)	60.73 ± 5.29	58.63 ± 8.19	0.177
FBG (mmol/L)	5.46 ± 0.89	5.67 ± 1.22	0.063
TG (mmol/L)	1.45 ± 1.44	2.53 ± 1.43	0.026
TC (mmol/L)	4.08 ± 1.37	4.33 ± 1.01	0.364
HDL-C (mmol/L)	0.91 ± 0.24	0.89 ± 0.17	0.7
LDL-C (mmol/L)	2.48 ± 1.11	2.62 ± 0.74	0.511
ALT (U/L)	35.37 ± 18.24	48.17 ± 17.63	0.002
AST (U/L)	31.30 ± 10.31	39.00 ± 28.00	<0.001
Cr (μmol/L)	69.49 ± 13.97	68.13 ± 12.60	0.646
UA (μmol/L)	356.67 ± 96.84	394.04 ± 84.07	0.068
BUN (mmol/L)	5.35 ± 2.64	4.79 ± 1.34	0.033

BMI, body mass index; SBP, systolic blood pressure; DBP, diastolic blood pressure; BPM, beat per minute; HTN, hypertension; DM, diabetes mellitus; EF, ejection fraction; FBG, fasting blood glucose; ALT, alanine aminotransferase; AST, aspartate aminotransferase; TG, triglyceride; TC, total cholesterol; HDL-C, high-density lipoprotein cholesterol; LDL-C, low-density lipoprotein cholesterol; Cr, creatinine; UA, uric acid; BUN, blood urea nitrogen.

### Diversity in the gut microbiome between the two groups

**Figure 1:**
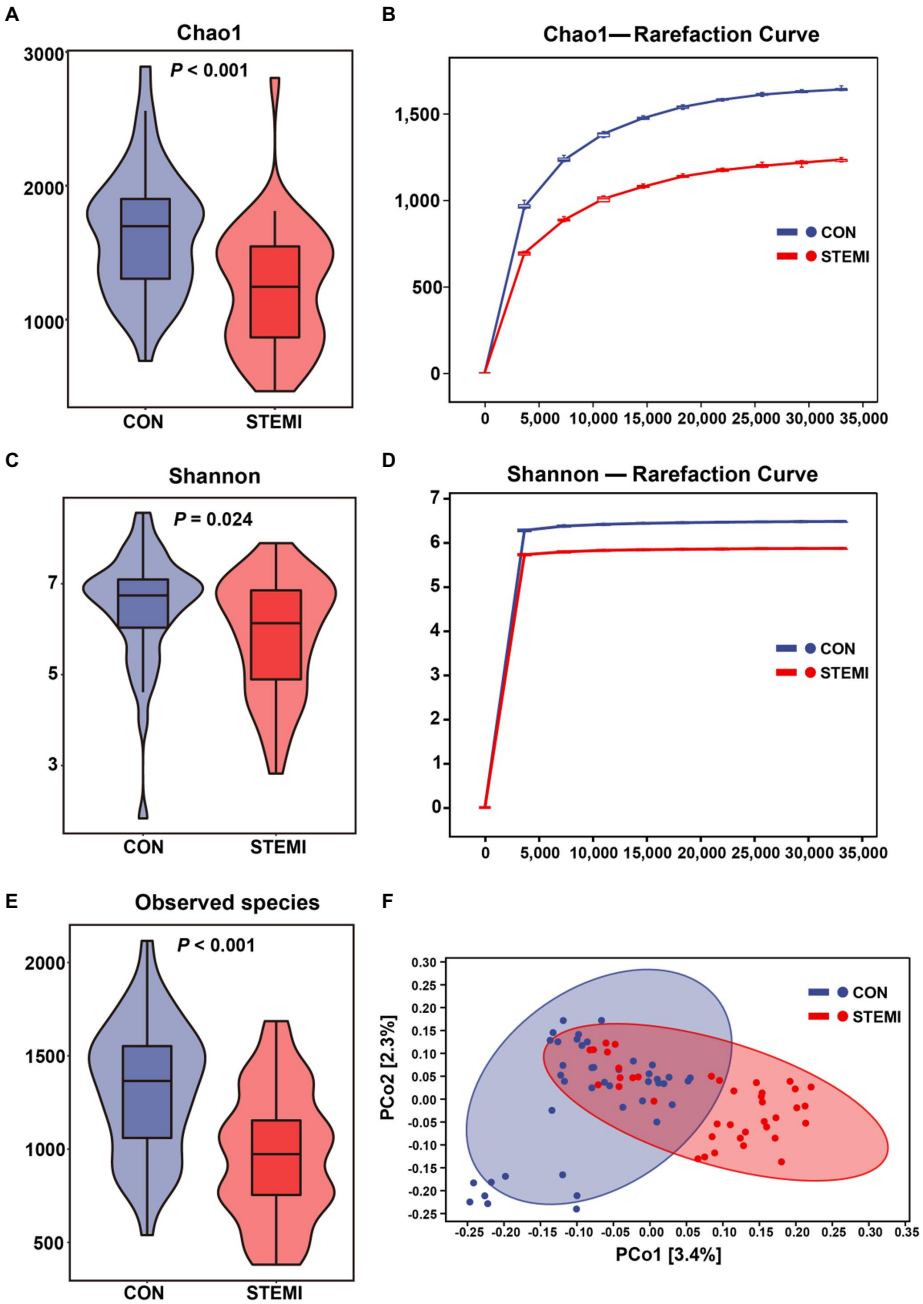
Physiological and microbial diversity in the two groups. **(A)** Chao1 index. **(B)** Chao1 curves for each group. **(C)** Shannon index. **(D)** Shannon curves for each group. **(E)** Observed species. **(F)** PCoA based on Jaccard distances of the physiological indexes among the two groups.

The microbial diversity was measured based on the α and β diversity analysis. Chao1 and Shannon indexes were used to quantify α diversity, and results revealed a significantly low α diversity in patients in the case group compared to the controls group (Figures 1A,B). Rarefaction analysis was performed for Chao1 and Shannon indexes of α diversity. The results confirmed a significant decrease in estimated observed operational taxonomic unit (OTU) richness in patients in the case group compared to the control group (Figures 1C,D). The score plot based on Jaccard distances and Principal coordinates analysis (PCoA) analysis for β diversity showed that the two groups were separated, and an approximately symmetrical distribution was observed between the two groups (Figure 1E). Venn diagrams shown in Supplementary Figure S1 further displaying the overlaps between groups showed that 6,883 of the total 47,122 ASVs were shared between the two groups, and 22,224 of 29,107 ASVs were unique for the controls and 18,015 of 24,898 ASVs were unique for the STEMI patients. The results consistently indicated that the ASVs level in the STEMI group was significantly lower than that in the control group.

### Taxonomic alterations in the gut microbiome between the two groups

**Figure 2:**
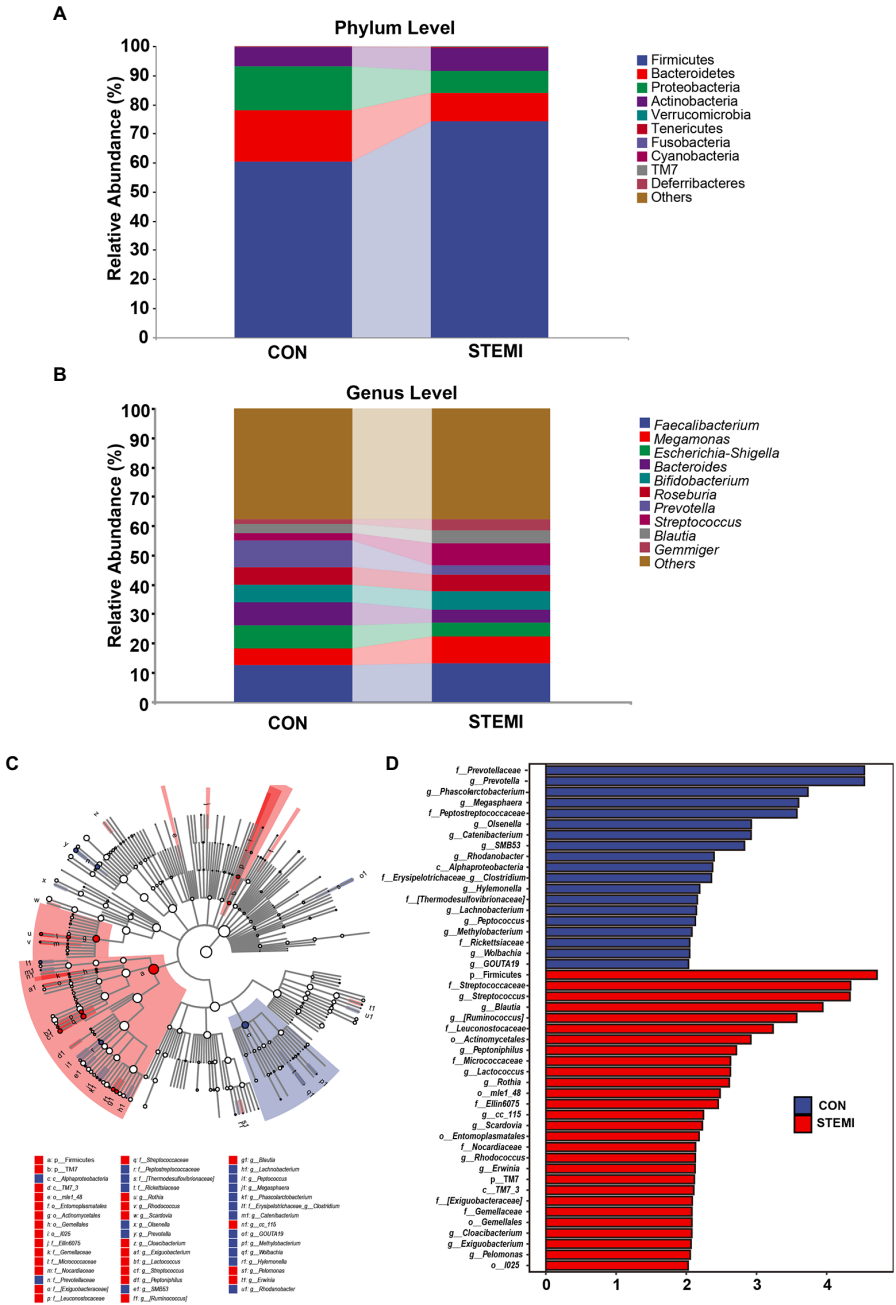
Taxonomic alterations in the patient and control groups. **(A)** Composition of gut microbiome communities at the phylum level. **(B)** Composition of gut microbiome communities at the genus level. **(C)** Cladogram and **(D)** Histogram of the linear discriminant analysis effect size (LEfSe) method (LDA > 2, *p* < 0.05) for differentially abundant gut microbiome between the two groups. Blue and red colors represent gut microbiome that were significantly overrepresented in the control groups and STEMI patients, respectively.

**Figure 3:**
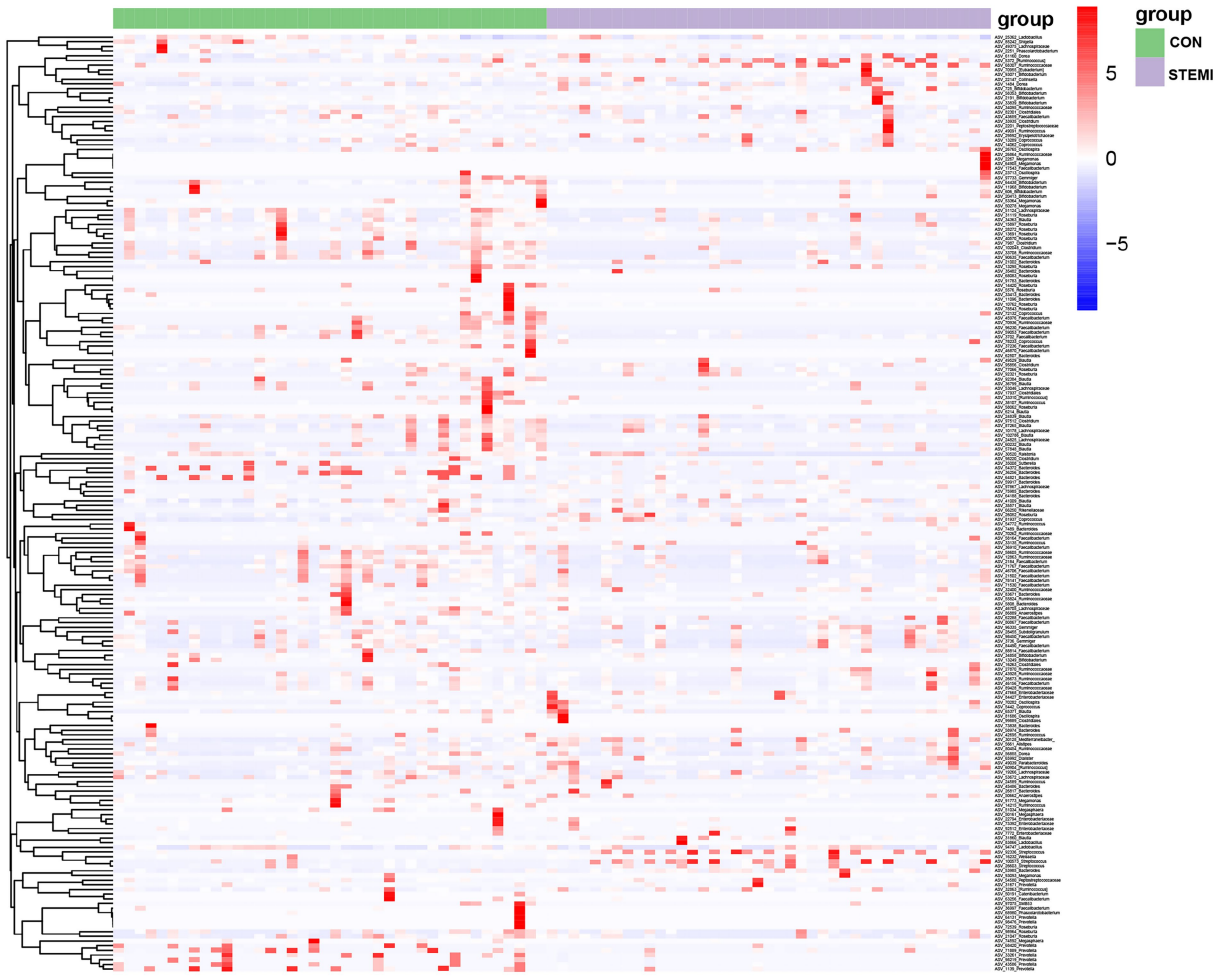
Heatmap of the correlations between the top 200 most abundant ASVs (hierarchically clustered).

At phylum level, Firmicutes, and Bacteroidetes were predominantly detected in the case and the control groups. The patients in the case group had a significantly high abundance of Firmicutes (74.08%) compared to the control group (60.49%). Whereas a significantly low abundance of Bacteroides (10.01%) and Proteobacteria (7.55) was observed in the patients in the case group compared to the control group (17.60 and 14.82%, respectively; Figure 2A). At genus level, the case group showed a significantly high abundance of genus *Streptococcus* (7.55%), *Megamonas* (9.55%), and *Gemmiger* (3.70%) compared to the control group (2.50, 5.46, and 1.44%, respectively). As shown in Figure 2B, the case group had a significantly low abundance of *Bacteroides* (4.23%) and *Prevotella* (2.91%) compared to the control group (8.05 and 9.05%, respectively). In previous studies Streptococcus was also associated with systemic inflammation and STEMI but so was Bacteroides (Zhou et al., 2018; Kwun et al., 2020), in apparent contradiction with our results. However, when analyzing the results on an ASV level, as shown further below (Figure 3), this apparent contradiction was resolved. The taxonomy of the gut microbiome was compared using LEfSe analysis to find relevant biomarkers. A total of 47 clades were screened in the stool sample based on a LDA threshold score of 2 (Figure 2C). Compared to the control group, significant (*p* < 0.05) decrease in 19 clades, including *Prevotellaceae (f)*, *Prevotella (g)*, *phasolarctobacterium (g)*, *Megasphaera (g)* and *Peptostreptococcaceae (f)* were observed in the patients in the case group. A significant (*p* < 0.05) increase in 28 clades, including *Firmicutes (f), Streptococcaceae (f), Streptococcus (g), Blautia (g),* and *[Ruminococcus] (g),* were observed in the case group, compared to the control group. Moreover, combined with the data in Supplementary Table S1, the heatmap of the correlations between the top 200 most abundant ASVs (hierarchically clustered) showed a clear separation between the two groups with a downward trend in the STEMI group (Figure 3). Specifically, 6 ASVs (ASV 33261, 98,219, 71,889, 68,420, 43,586, 1,139) belonging to *Prevotella copri* species, 3 ASVs (ASV 54372, 36,256, 64,821) belonging to *Bacteroides uniformis* species, 4 ASVs (ASV 15897, 28,272, 13,691, 40,570) belong to *Roseburia* genus decreased in the STEMI group; However, ASV 59917 and ASV 53985 belonging to *Bacteroides vulgatus* species were very strongly positively associated with STEMI. Likewise, the ASV 31860 belonging to *Blautia wexlerae* species is diametrically opposed to ASV 24839 belong to *Blautia massiliensis* species. Consistent with the analysis of genus level, an increased abundance of 5 ASVs (ASV 5372, 68,307, 92,336, 26,003, 100,573) belonging to *[Ruminococcus]* and *Streptococcus* genus, respectively, were also observed in the STEMI group. These results suggest that the alteration in the abundance of *Streptococcus and Prevotella* were the characteristic changes in the gut microbiome in young male patients with STEMI.

### Correlation between clinical parameters and changes in the microbiome

**Figure 4:**
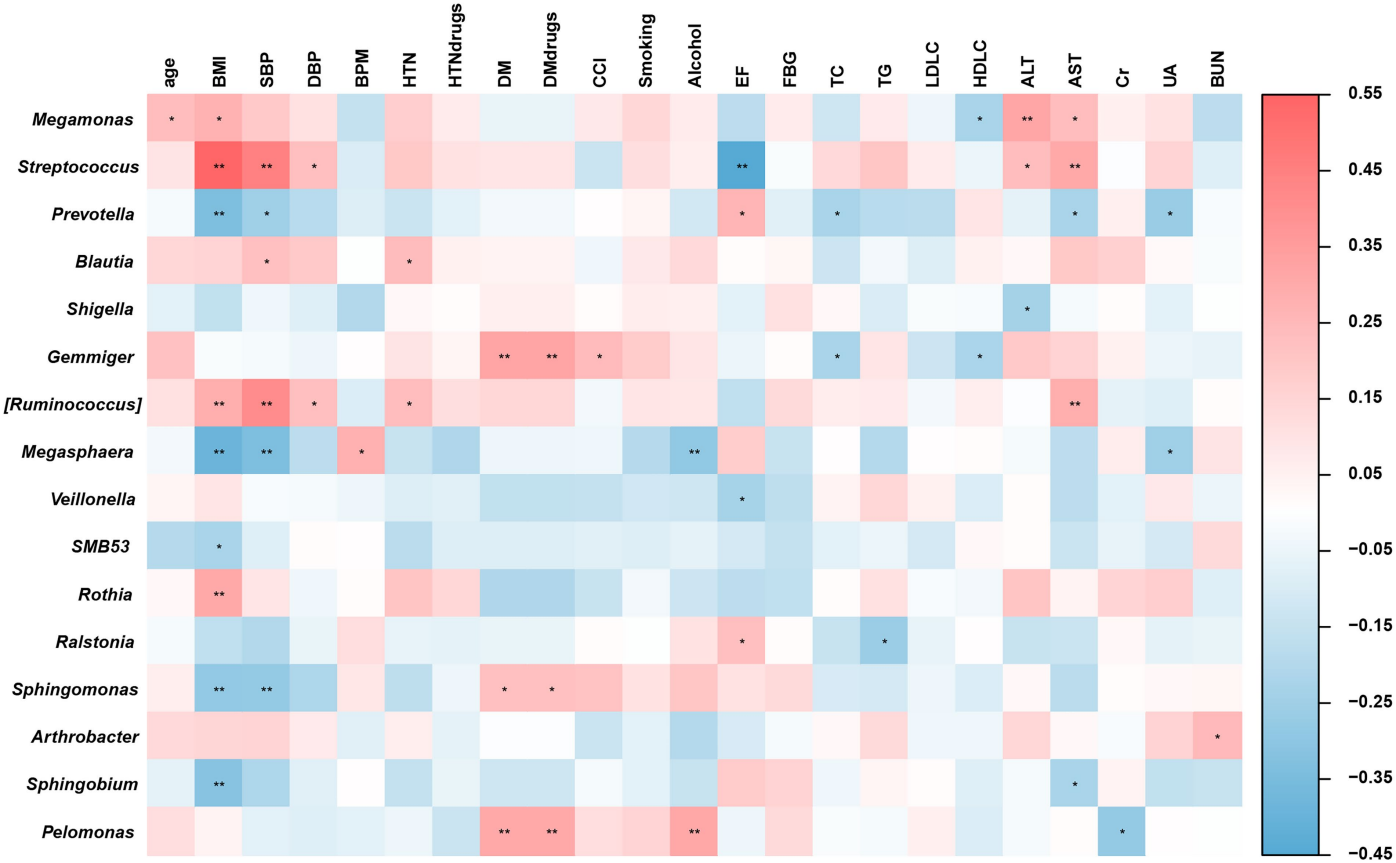
Heatmaps of Spearman’s correlations between altered gut microbiome and physiological indices. *p < 0.05; **p < 0.01.

Based on the ecological enterotype gradient by principal coordinates analysis of inter-individual differences in the microbiome profile, BMI, AST and SBP were identified to be contribute immensely to gene-level microbiome among all the clinical parameters (Supplementary Figure S2). Then spearman’s rank correlation analysis was performed on the clinical parameters and specific microbiomes between the two groups. As is shown in Figure 4, BMI and SBP were significantly (*p* < 0.05) positively correlated with *Streptococcus* and *[Ruminococcus]*. Further, BMI and SBP were negatively correlated with *Prevotella* and *Megasphaera* (*p* < 0.05). Both HTN history and AST were significantly (*p* < 0.05) positively correlated with *Streptococcus*, *Megamonas,* and *[Ruminococcus]*. A significant (*p* < 0.05) negative correlation was observed between *Prevotella* and AST. However, there was no significant difference in EF between the case and control groups. A significant (*p* < 0.05) negative correlation was observed between EF and *Streptococcus.* A significant (*p* < 0.05) positive correlation was observed between EF and *Prevotella.* The Prevotella enterotype gradient analysis further confirmed this phenomenon. These results suggest that alterations in the microbiome compositions and abundances of microbial groups specifically *Prevotella* and *Streptococcus*, could indicate biochemical and metabolic changes in young male patients with STEMI.

### Combined prediction model of the gut microbiome and clinical variables

**Figure 5:**
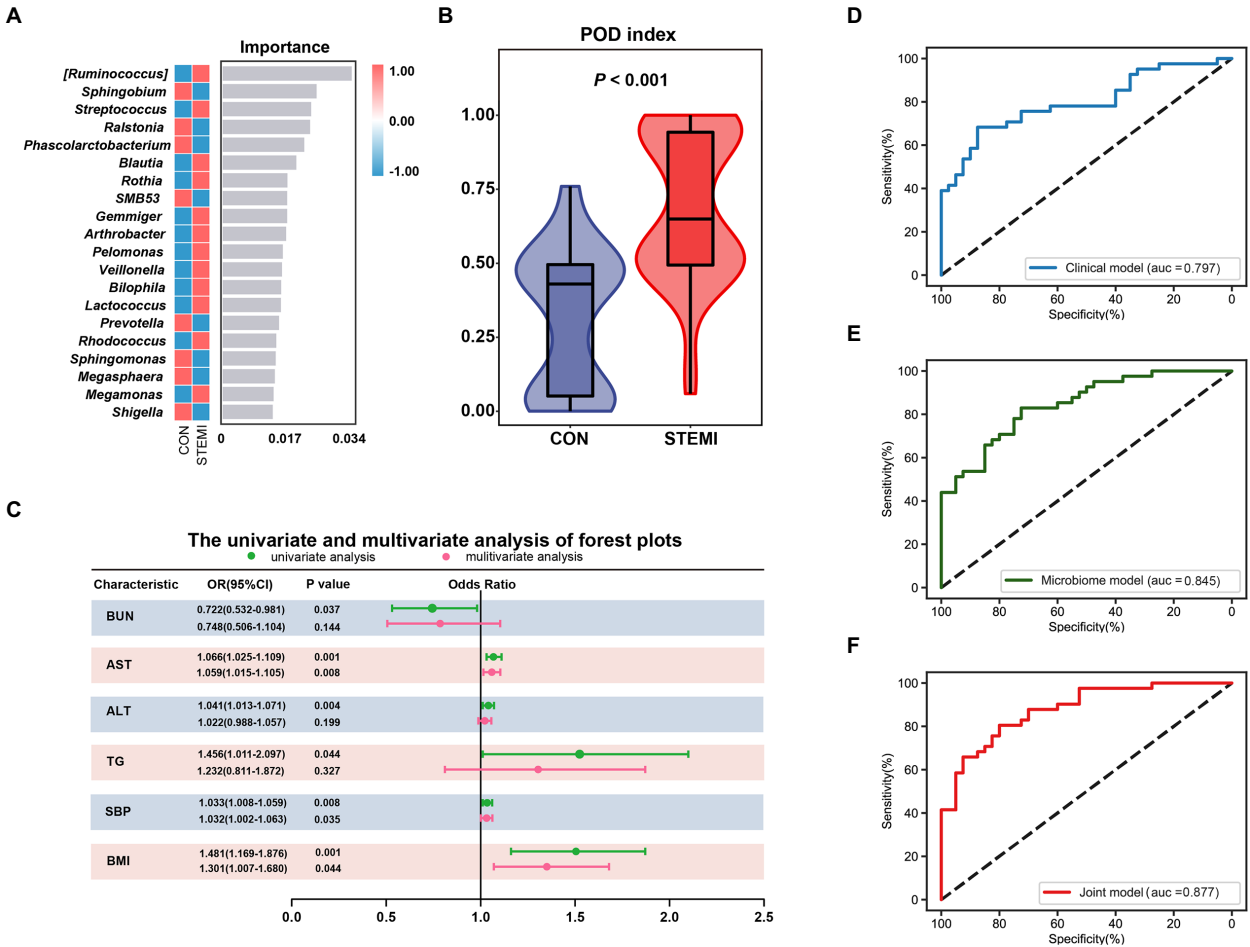
Combined prediction model of gut microbiome and clinical variables. **(A)** The top 20 bacteria belong to the genus level based on the random forest analysis. **(B)** Comparison of the POD of gut microbiome between the two groups. **(C)** Candidate variables for clinical model development were presented as forest plots. The AUCs for the diagnostic performances of the clinical model **(D)**, the gut microbiome model **(E)**, and combined model **(F).**

The relative abundance of 20 gut microbiome markers was detected using random forest analysis. Logistics regression analysis was used to calculate the probability of disease (POD) index based on these screened 20 gut microbiome markers (Figure 5A). As shown in Figure 5B, a significantly (*p* < 0.001) high POD index was observed in the case group compared to the control group. The candidate clinical parameters intended to be incorporated into the clinical model were based on the univariate and multivariate logistic regression analysis (Supplementary Table S2). Finally, three clinical parameters such as BMI (*p* < 0.05), SBP (*p* < 0.05), and AST (*p* < 0.01) were included in the model (Figure 5C). However, the clinical model barely achieved an AUC value of 0.797 (Figure 5D). The AUC value for the microbiome model based on the POD index was 0.845. These results indicate that the microbiome model was superior to the clinical model (Figure 5E). A weak to moderate correlation was observed between the gut microbiome markers and clinical parameters. The multicollinearity between gut microbiome markers and clinical parameters was analyzed and excluded prior to their incorporation into the combined model. As expected, the performance of the combined model was optimum. The performance of the combined model was better compared to the clinical model (AUC: 0.877 vs. AUC: 0.797) or the microbiome model (AUC: 0.877 vs. AUC: 0.845; Figure 5F) individually.

### Nomogram construction and validation of subjects

**Figure 6:**
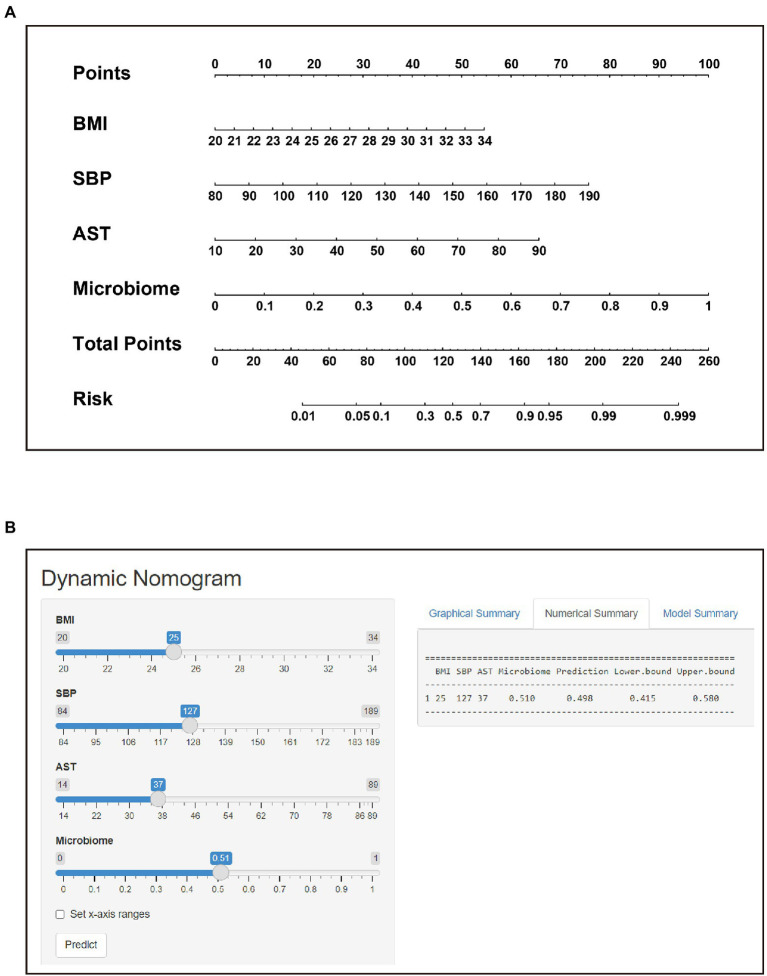
Nomogram and its corresponding web page. **(A)** Construction of the nomogram based on BMI, SBP, AST and Microbiome to assign the probability of developing STEMI. **(B)** Web-based risk calculator (Dynamic Nomogram (shinyapps.io)) to predict incidence rate of STEMI.

**Figure 7:**
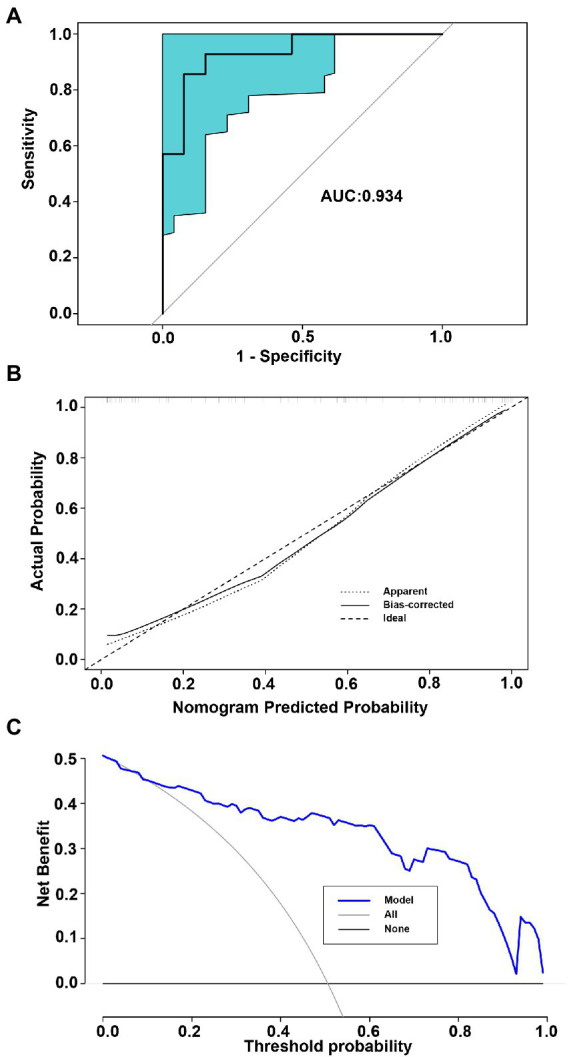
Internal validation for the model performance. **(A)** The AUCs for stratified K-fold cross-validation (*K* = 3). **(B)** Calibration curves of the nomogram for predicting probability of developing STEMI. The x-axis represents the nomogram-predicted probability, whereas the y-axis represents the actual probability. **(C)** Net benefit of using a model to diagnose STEMI compared with the strategies of “treating all” or “treating none” for different decision thresholds.

The POD index of the gut microbiome and the screened clinical parameters were combined to construct a nomogram (Figure 6A). The diagnostic index (DI) was calculated using the following formula: −15.464 + 0.227 × BMI (kg/m^2^) + 0.040 × SBP (mmHg) + 0.048 × AST (U/L) + 5.803 × POD of microbiome. A web-based dynamic nomogram was constructed to predict the risk of STEMI in patients (https://lucky-lmc-nomogram.shinyapps.io/DynNomapp/, Figure 6B). The diagnostic performance of the nomogram was evaluated using the consistency index (C-index), and the C-index of the nomogram was 0.911. For the internal validation, the stratified K-fold cross-validation (*K* = 3) was as follows: AUC was 0.934 (Figure 7A). The calibration curves of the model showed good consistency between the actual and predicted probabilities (Figure 7B). The DCA showed that the model curve was far from the two reference curves (all and none), indicating a high net clinical benefit (NCB) for clinical use (Figure 7C). These results show that the nomogram model based on the gut microbiome and clinical parameters has good diagnostic efficacy and practicability.

## Discussion

In this study, we performed a comprehensive analysis of the gut microbiome and clinical parameters between age and gender-matched patients with STEMI and non-CAD males. Our results revealed a significant difference in the α and β diversity between the two groups. The gut microbiome was significantly altered between the two groups, specifically alterations in the abundance of *Streptococcus* and *Prevotella*. Based on these results, prognostic models were established. The model was internally validated and optimized by combining it with clinical parameters. Our study is novel in the following aspects: We have performed comprehensive analyses of biodiversity to alterations at taxonomy levels in the gut microbiome. We have used a robust methodology to identify prognostic factors with internal validations and established an easy-to-use, non-invasive risk prediction model. The model constructed based on a combination of the gut microbiome and clinical parameters had satisfactory diagnostic efficacy and practicability in predicting risk in young male patients with STEMI.

The richness and diversity of the gut microbiome are important for maintaining the homeostasis and performance of the body. Multiple studies have demonstrated that dysbiosis in the gut microbiome is associated with various diseases, including CAD (Schirmer et al., 2018; Zhou et al., 2018, 2022). Previous studies have demonstrated a decrease in the richness and diversity of the gut microbiome in patients with hypertension or heart failure (Li et al., 2017; Luedde et al., 2017). Similarly, our results showed a decrease in microbiome diversity in young male patients with STEMI. Further, the alterations in the genus level were analyzed, and the results showed a reduction in the abundance of *Prevotella* and an increase in the abundance of *Streptococcus*. Jie et al. performed a metagenomic association analysis, and the results showed a close association between an increase in the abundance of *Streptococcus* spp. and atherosclerosis (Jie et al., 2017). A previous study has also demonstrated that oral *Streptococcus* causes infective endocarditis and aortic valve lesions, which eventually progress to acute myocardial infarction (AMI), characterized by chest pain, dyspnea, and heart failure (Sugi et al., 2015). An increase in the abundance of *Streptococcus* was also detected in coronary atheromatous plaque specimens of AMI patients (Joshi et al., 2021). Therefore, our results were consistent with previous studies. We would like to speculate that the translocation of periodontal and intestinal *Streptococcus* to the coronary atheroma *via* systemic circulation may accelerate the development of STEMI in young males. Various studies have shown that the role of *Prevotella* is controversial. Clinical studies have shown that an increase in the abundance of *Prevotella* in mucosal sites was associated with local inflammatory and systemic metabolic diseases, including rheumatoid arthritis, diabetes, and hypertension (Larsen, 2017; Li et al., 2017; Yang et al., 2021). However, Yin et al. reported a decrease in the abundance of *Prevotella* in patients with large artery atherosclerotic ischemic stroke/ transient ischemic attack compared to the matched control group (Yin et al., 2015). We used multiple analysis methods, including composition, LEfSe, and random forest analysis, and the results revealed that *Prevotella* was consistently down-regulated in young male patients with STEMI. This indicates that *Prevotella* conferred protection against the occurrence of STEMI in young male patients. Moreover, our results show an inverse relationship between *Prevotella* and obesity, consistent with previous findings (Ley et al., 2006). Most patients in our study were overweight or obese (BMI exceeded 25 kg/m^2^ in 75% of patients). In obese patients, the decrease in the abundance of *Prevotella* could not restrain the upregulation inflammatory factors levels, including endotoxin and IL-6 (Claesson et al., 2012). This activated inflammatory response ultimately ruptures the atherosclerotic plaque. Consistent with other studies, the LEfSe or random forest analysis performed in our study also show alterations in the abundance of *[Ruminococcus]*, *Megasphaera,* and *Megamonas* (van den Munckhof et al., 2018; Liu et al., 2020; Zheng et al., 2020). Furthermore, it is crucial to focus on how mediation of these characterized gut microbiome exerts effect on STEMI. Current potential treatment opportunities, such as the administration of probiotics, prebiotics and fecal transplantation, may be appropriate treatment options for STEMI. Studies have demonstrated that probiotic *Lactobacillus* and *rhamnosus* GR-1 can attenuate postinfarction remodeling and heart failure in rats and humans (Gan et al., 2014; Moludi et al., 2021). Fecal transplantation from hypertensive human donors into germ-free mice showed increased blood pressure in the mice (Li et al., 2017). A large retrospective cohort study demonstrated that transplantation of fecal content into *Clostridioides* difficile patients increased the risk of myocardial infarction (Dawwas et al., 2022). Thus, our results may provide a new strategy for the prevention, treatment, and management of STEMI based on the gut microbiome.

For decades, common clinical parameters and novel biomarkers, including lipid metabolites and exosomal miRNA, have been used to predict AMI. However, the predictive value of these biomarkers is moderate (Eichler et al., 2007; Su et al., 2020; Liu et al., 2022). In our study, the analysis of the clinical parameters revealed a correlation between parameters like BMI, SBP, AST, and STEMI in young males. Our results are different from Sagris et al. study, where tobacco use, dyslipidemia, and diabetes mellitus were the risk factors for young adults (age < 45 years) with AMI (Sagris et al., 2022). In contrast, we enrolled young (average age < 40 years) male patients with STEMI male during the active phase. The discrepancies in the results between the studies could be due to differences in the enrollment time frames and study populations. Mounting evidence has indicated that obesity, blood pressure, and AST were significantly associated with the development and prognosis of patients with STEMI (Mentias et al., 2017; McEvoy et al., 2019; Konijnenberg et al., 2020). Unfortunately, the ability of the clinical prediction model to discriminate based on these three parameters was not satisfactory, with an AUC value of 0.797. In our study population, a strong correlation was observed between multiple gut microbiomes and STEMI. Hence, a microbiome prediction model was established based on 20 genera, including *Streptococcus*, *Prevotella*, *[Ruminococcus]*, *Megasphaera,* and *Megamonas.* The performance of the microbiome prediction model was significantly better compared to the clinical model. These results show that the gut microbiome plays an important role in STEMI in young males. Despite its impressive performance, the prediction model based on the gut microbiome alone may not be sufficient due to the complex nature of the disease. Therefore, we incorporated the clinical parameters and gut microbiome into a combined model to optimize its performance in predicting the disease. As expected, the combination of clinical parameters and gut microbiome enhanced the predictive ability of the model with an AUC value of 0.877. To improve its clinical practicability, we constructed a nomogram and accessible web page to visualize the model and provide an easily accessible individual prediction. A previous study constructed a prediction model based on the 24 genera and 72 serum metabolites for CAD with an AUC value of 0.897 (Liu et al., 2019). However, this model was relatively complex and consuming as it required invasive blood sampling to analyze extensive indices, and the cost of metabolomics analysis was high. This may limit its application in clinical settings. On the contrary, our combined model was advantageous due to its simple and non-invasiveness nature, as well as higher accuracy in predicting STEMI in young males. Further, our model could achieve predictive ability equivalent to other models with fewer parameters and did not require invasive sampling.

However, our study has a few limitations. First, our study is a single-center retrospective analysis which could lead to potential bias. However, consecutive patients were enrolled to reduce selection bias as much as possible. Further, the analysis performed at single-center guaranteed quality control. However, additional studies with larger sample sizes are needed to validate our results. Second, we did not analyze microbiome-related metabolites and associated pathways. Third, the sample size of our study was relatively small, which did not allow external validation. However, we used robust statistical methods such as bootstrap resampling to ensure good internal validity.

In conclusion, we enrolled age and gender-matched young male patients with STEMI and non-CAD males. Our analysis showed the alteration in microbiome diversity between the two groups, and the screened gut microbiome had significant predictive value. Further, a prediction model combining non-invasive clinical parameters and gut microbiome was established to predict the occurrence of STEMI in young males. Based on the underlying pathological mechanism for the early onset of STEMI, our results may provide new insights for predicting the occurrences of STEMI using fluid metabolites such as stool, urine, and saliva in the future.

## Data availability statement

The datasets presented in this study can be found in online repositories. The names of the repository/repositories and accession number(s) can be found at: NCBI - PRJNA883944.

## Ethics statement

The studies involving human participants were reviewed and approved by ethics committee of Tangdu Hospital of Air Force Medical University. The patients/participants provided their written informed consent to participate in this study.

## Author contributions

JH and YL: conception of design and analysis. ML, MW, TP, and WM: data collection and drafting of the manuscript. QW and XN: statistical analysis. LH, BQ, DG: manuscript review and revision. JG and LS: fecal sample collection. All authors contributed to the article and approved the final manuscript.

## Funding

This work was supported by the National Natural Science Foundation of China (nos. 82070385 and 82000350) and by Young Talent Fund of University Association for Science and Technology in Shaanxi, China (no. 20210305).

## Conflict of interest

The authors declare that the research was conducted in the absence of any commercial or financial relationships that could be construed as a potential conflict of interest.

## Publisher’s note

All claims expressed in this article are solely those of the authors and do not necessarily represent those of their affiliated organizations, or those of the publisher, the editors and the reviewers. Any product that may be evaluated in this article, or claim that may be made by its manufacturer, is not guaranteed or endorsed by the publisher.
