# Supplementary material for: Gut-microbiome-based predictive model for ST-elevation myocardial infarction in young male patients
Source: Front Microbiol. 2022 Dec 1;13:1031878. doi: 10.3389/fmicb.2022.1031878 (PMC9756097; doi:10.3389/fmicb.2022.1031878)
Supplement: Supplementary file 1 [file Data_Sheet_1.docx]

# Supplementary materials

**Gut-Microbiome-based predictive model for ST-elevation**

**myocardial infarction in young male patients**

*Mingchuan Liu ^1, †^, Min Wang ^2, †^, Tingwei Peng ^1, †^, Wenshuai Ma ^1, †^, Qiuhe Wang ^1^, Xiaona Niu ^1^, Lang Hu ^1^, Bingchao Qi ^1^, Dong Guo ^1^, Gaotong Ren ^1^, Di Wang^1^, Jing Geng ^1^, Liqiang Song ^2^, Jianqiang Hu ^1, *^, Yan Li ^1, *^*

**TABLE S2Ⅰ**Candidate variables for clinical model development

| **Variables** | **AUC** | **P values** | **95% CI** |
| --- | --- | --- | --- |
| Age | 0.62 | 0.064 | 0.496-0.743 |
| BMI | 0.722 | 0.001 | 0.612-0.832 |
| SBP | 0.673 | 0.008 | 0.551-0.794 |
| DBP | 0.576 | 0.24 | 0.451-0.701 |
| BPM | 0.457 | 0.502 | 0.329-0.585 |
| LDL-C | 0.566 | 0.305 | 0.440-0.692 |
| TC | 0.587 | 0.177 | 0.462-0.713 |
| TG | 0.644 | 0.026 | 0.523-0.764 |
| Cr | 0.486 | 0.828 | 0.359-0.613 |
| HDL-C | 0.438 | 0.335 | 0.310-0.565 |
| HTN | 0.595 | 0.141 | 0.471-0.719 |
| DM drugs | 0.536 | 0.574 | 0.410-0.662 |
| HTN drugs | 0.547 | 0.47 | 0.421-0.673 |
| DM | 0.536 | 0.574 | 0.410-0.662 |
| Comorbidity | 0.523 | 0.723 | 0.397-0.649 |
| Smoking | 0.567 | 0.297 | 0.442-0.693 |
| FBG | 0.62 | 0.063 | 0.498-0.742 |
| Alcohol | 0.534 | 0.6 | 0.408-0.660 |
| ALT | 0.702 | 0.002 | 0.588-0.815 |
| AST | 0.705 | 0.001 | 0.593-0.818 |
| EF | 0.44 | 0.355 | 0.312-0.568 |
| UA | 0.638 | 0.032 | 0.515-0.762 |
| BUN | 0.4 | 0.122 | 0.275-0.526 |


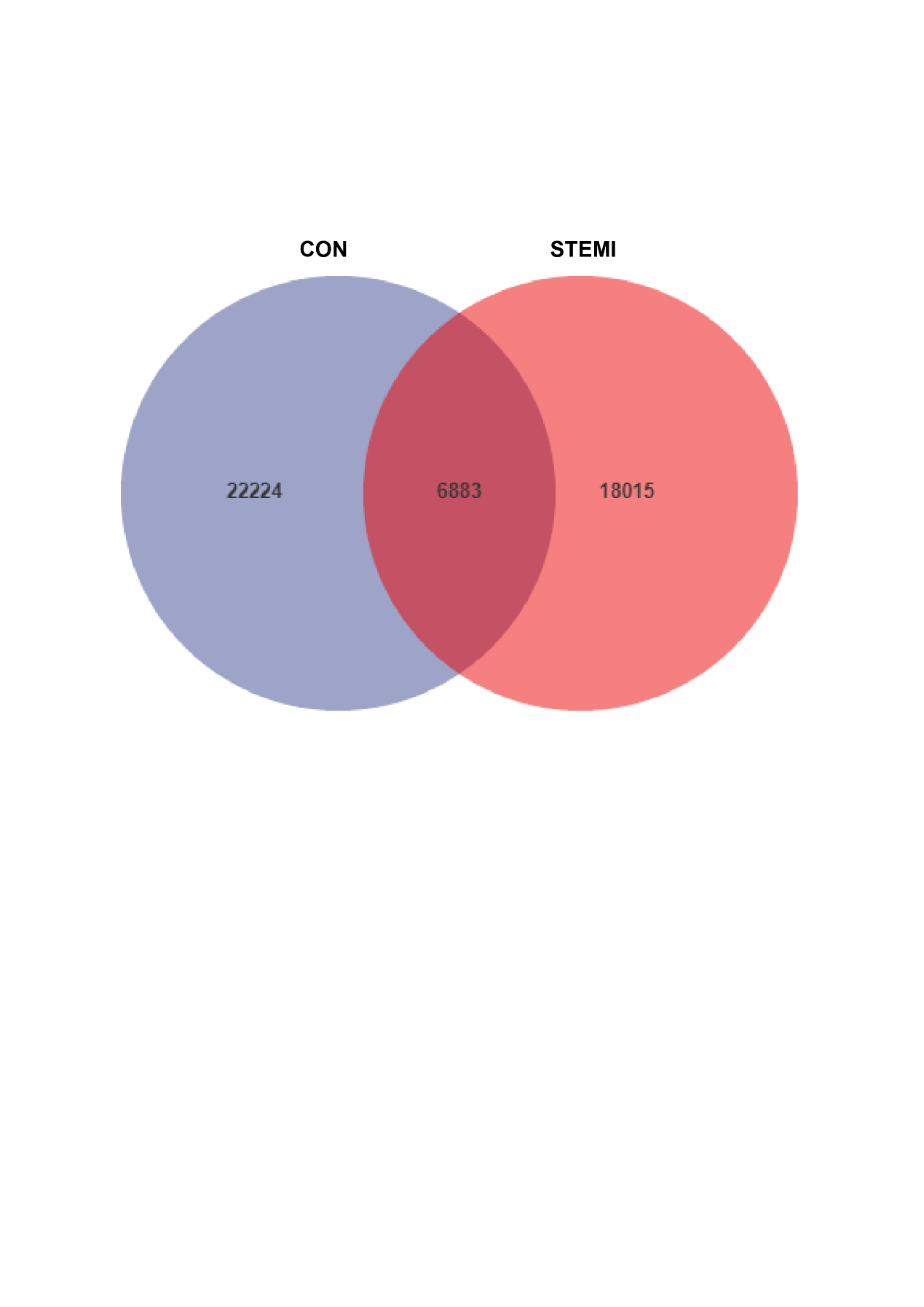


**FIGURE S1Ⅰ**Venn diagram displaying the overlaps between groups showing that 6883 of the total richness of 47122 ASVs were shared between the STEMI patients and the healthy controls.


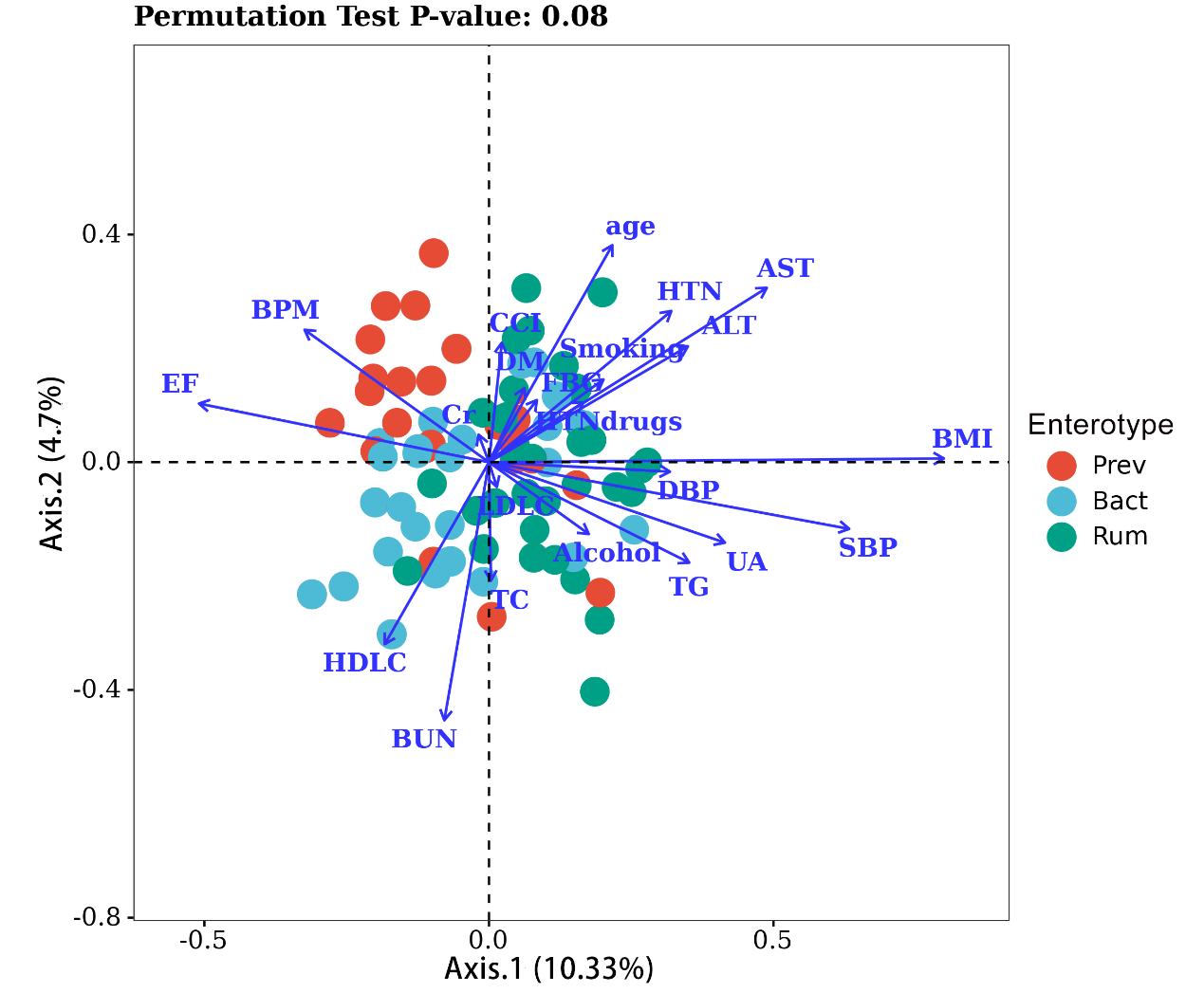


**FIGURE** **S2Ⅰ**Principal coordinates analysis of inter-individual differences (genus level Bray Curtis dissimilarity) in the microbiome profile.
